# Benefits of The Unforgettables: A Chorus for People With Dementia Together With Their Family Caregivers

**DOI:** 10.1093/geroni/igaa057.2838

**Published:** 2020-12-16

**Authors:** Mary Mittelman

**Affiliations:** NYU School of Medicine, New York, New York, United States

## Abstract

The Unforgettables was founded in 2011 for people with dementia and their family caregivers. We hypothesized that singing and rehearsing together would providing an opportunity for people in the early and moderate stages of dementia and their family caregivers to share a normative, stimulating and social activity. Pilot study results showed that quality of life and communication with the other member of the dyad improved for people with dementia; quality of life and, social support, communication and self- esteem improved for caregivers. Moreover, people with dementia learn new songs for every performance, suggesting that this activity may slow cognitive decline. The chorus continues to rehearse and perform, and now has approximately 100 members in two locations in NYC. These findings support the recommendations of the Global Council on Brain Health by underscoring the many benefits of music performance in enhancing social engagement and providing joy to participants and the community.

